# GLP-1 Agonists in Cardiovascular Diseases: Mechanisms, Clinical Evidence, and Emerging Therapies

**DOI:** 10.3390/jcm14196758

**Published:** 2025-09-24

**Authors:** Han-Mo Yang

**Affiliations:** Division of Cardiology, Department of Internal Medicine, Seoul National University Hospital, Seoul 03080, Republic of Korea; hanname@gmail.com; Tel.: +82-2-2072-4184

**Keywords:** GLP-1 receptor agonists, cardiovascular diseases, type 2 diabetes, atherosclerosis, heart failure, stroke, vascular dementia, dual GLP-1/GIP agonists, cardiovascular outcome trials, inflammation

## Abstract

Glucagon-like peptide-1 (GLP-1) receptor agonists now serve as therapeutic agents for cardiovascular diseases (CVDs) beyond their original use for treating type 2 diabetes mellitus (T2DM). This review combines molecular mechanisms with clinical evidence to demonstrate how GLP-1 agonists help lower cardiovascular risk for conditions, including atherosclerosis, heart failure, stroke, and vascular dementia. These agents produce multiple beneficial effects, which include anti-inflammatory action along with anti-atherogenic effects, endothelial-protective benefits, and cardioprotective actions to minimize major adverse cardiovascular events (MACEs). GLP-1 agonists achieved substantial reductions in myocardial infarction, stroke, cardiovascular mortality, and heart failure events according to major cardiovascular outcome trials (CVOTs). Recent research, notably the pivotal SELECT trial, has confirmed their suitability for non-diabetic subjects with obesity and established CVD. New drug delivery methods and dual GLP-1/glucose-dependent insulinotropic polypeptide (GIP) agonists demonstrate potent efficacy, with tirzepatide showing significant MACE reduction in its own CVOT. However, significant challenges related to high cost, long-term safety uncertainties, and implementation barriers remain, requiring a balanced perspective. The review presents both mechanistic data and clinical evidence to demonstrate how GLP-1 agonists function as vital cardiovascular medications and outlines future research directions to address critical evidence gaps and maximize their therapeutic effectiveness.

## 1. Introduction

Cardiovascular diseases (CVDs) continue to be the main cause of global mortality, resulting in an estimated 18.6 million deaths annually, with age-standardized death rates showing persistent elevation despite advances in preventive therapies [1,2]. Metabolic disorders, particularly type 2 diabetes mellitus (T2DM) and obesity, contribute significantly to this burden, affecting over 460 million people worldwide with diabetes and creating a substantial interplay between metabolic and cardiovascular pathophysiology [1,2]. The convergence of T2DM and CVD has led to the creation of drugs that can manage both glucose levels and cardiovascular risks, and Glucagon-like peptide-1 (GLP-1) receptor agonists are considered a significant advancement [3,4]. The incretin system, comprising GLP-1 and glucose-dependent insulinotropic polypeptide (GIP), plays a fundamental role in postprandial glucose homeostasis. The GLP-1 receptor agonists (e.g., liraglutide, semaglutide, dulaglutide, and the dual GLP-1/GIP agonist tirzepatide) were first developed for T2DM, and they act by mimicking the incretin hormone GLP-1, which increases glucose-dependent insulin secretion, decreases glucagon secretion, and prolongs gastric emptying [5]. Beyond glycemic control, these agents have been investigated for their pleiotropic anti-inflammatory, anti-atherogenic, and cardioprotective effects, addressing unmet needs in non-diabetic populations with obesity and CVD. In particular, the discovery of GLP-1 receptors in cardiovascular tissues opened new therapeutic possibilities beyond glycemic control. In addition to the glycemic control, these agents have shown considerable cardiovascular advantages that have been confirmed by large-scale cardiovascular outcome trials (CVOTs) that have shown reductions in major adverse cardiovascular events (MACE), including myocardial infarction (MI), stroke, and cardiovascular death [6,7]. 

The escalating prevalence of CVD, coupled with increasing T2DM and obesity rates, makes it important to find new ways of reducing the residual cardiovascular risk in patients treated with conventional therapies, including statins, antihypertensives, and antiplatelet agents [8]. GLP-1 agonists bridge this therapeutic gap by having pleiotropic effects, including anti-inflammatory, anti-atherosclerotic, endothelial-protective, and cardioprotective actions [9,10]. Pivotal studies, such as the STEP-HFpEF and SELECT trials, have now expanded their indication to heart failure with preserved ejection fraction (HFpEF) and to non-diabetic populations with obesity and CVD, respectively [11,12]. In addition, there is some evidence that they may also be useful in cerebrovascular diseases, such as stroke and vascular dementia, given their common underlying pathophysiology with CVD [13]. Dual GLP-1/GIP agonists, such as tirzepatide, have been introduced, and they offer greater weight loss and potential cardiovascular advantages [14].

While several reviews on this topic exist, this article aims to provide a distinct, up-to-the-minute synthesis that addresses the needs identified by recent expert commentary. This review is differentiated by (1) incorporating the very latest 2024 and 2025 clinical trial data (e.g., SURPASS-CVOT, SOUL), ensuring maximal timeliness (Table 1); (2) offering a uniquely broad and integrative scope that connects molecular mechanisms not only to traditional Atherosclerotic Cardiovascular Disease (ASCVD) and heart failure but also extends the discussion to the emerging and clinically important fields of stroke and vascular dementia; and (3) presenting a dedicated critical appraisal of evidence gaps, safety controversies, and real-world implementation challenges, providing a balanced perspective that is essential for clinicians. This review brings together a detailed overview of the mechanisms, clinical evidence and upcoming uses of GLP-1 agonists in CVDs, focusing on their use in the prevention, treatment, and future treatment possibilities, including dual agonists. It also discusses the challenges, such as cost, access and long-term safety, and also examines the potential for precision medicine strategies to enhance their impact on the global CVD burden [15,16].

## 2. Mechanisms of GLP-1 Agonists in Cardiovascular Protection

**Figure 1:**
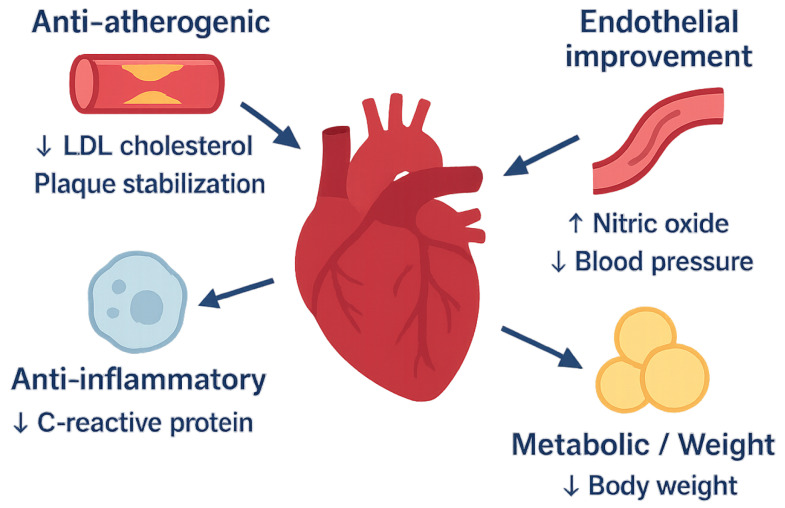
Principal mechanisms by which GLP-1 receptor agonists confer cardiovascular protection. A conceptual infographic summarising the multi-system actions of GLP-1 receptor agonists (GLP-1RAs). Central activation of the GLP-1 receptor in cardiometabolic tissues translates into the following: (1) anti-atherogenic effects (lower low-density lipoprotein cholesterol [LDL-C], plaque stabilisation); (2) endothelial and vascular improvement (enhanced endothelial nitric-oxide synthase activity, modest blood-pressure reduction); (3) anti-inflammatory actions (down-regulation of C-reactive protein, tumour necrosis factor-α, interleukin-6, and NLRP3 inflammasome activity); (4) metabolic/weight-loss benefits that improve insulin sensitivity and haemodynamic load. Together, these pathways converge to reduce major adverse cardiovascular events (MACE) and heart-failure outcomes documented in large outcome trials.

GLP-1 receptor agonists improve cardiovascular outcomes by multiple mechanisms that interact with each other and the pathophysiological processes of CVD. GLP-1 receptors are present in vascular endothelium, cardiomyocytes, vascular smooth muscle cells (VSMCs), immune cells, and the central nervous system, thus enabling multiple physiological effects (Figure 1 and Table 2) [23]. This section discusses these mechanisms in detail, integrating cell-specific signalling pathways within each functional domain and carefully distinguishing between preclinical evidence and data from human studies, and finally concluding with a discussion of how these pathways interact to produce a cohesive protective effect. 

### 2.1. Anti-Atherogenic Effects

Atherosclerosis, the primary driver of CVD, involves lipid accumulation, inflammation, and plaque formation in arterial walls [35]. GLP-1 agonists mitigate this process through direct actions on vascular and immune cells, in addition to their systemic metabolic effects.

#### 2.1.1. Lipid Metabolism Modulation

In human clinical studies, Through lipid metabolism modulation GLP-1 agonists decrease low-density lipoprotein cholesterol (LDL-C) levels and triglycerides along with apolipoprotein B but increase high-density lipoprotein cholesterol (HDL-C) [36]. Mechanistically, GLP-1 agonists appear to work by blocking VLDL production in the liver and activating lipoprotein lipase which results in better lipid profiles [36]. For example, the clinical trials showed Semaglutide lowered LDL-C by 5–10% and liraglutide reduced apolipoprotein B by 8% [37].

#### 2.1.2. Plaque Stabilization

Preclinical studies on GLP-1 agonists show their ability to decrease macrophage infiltration while inhibiting foam cell formation and reducing matrix metalloproteinase-9 (MMP-9) expression, which helps create fibrous caps for plaque stability [24]. Specifically, in ApoE-knockout mice, liraglutide decreased atherosclerotic lesion size by 30% by reducing monocyte chemoattractant protein-1 (MCP-1) expression. [25]. Similarly, semaglutide decreased plaque progression in LDLR-deficient mice by downregulating pro-inflammatory chemokines [24].

#### 2.1.3. Signaling in VSMC

The abnormal proliferation and migration of VSMCs are key events in plaque growth and instability. GLP-1 agonists directly counter this. Upon receptor activation in VSMCs, intracellular levels of cyclic AMP (cAMP) increase, which in turn reduces the activity of pro-proliferative pathways, such as nuclear factor-kappa B (NF-κB) and mitogen-activated protein kinase (MAPK) pathways [9]. The inhibition of these critical transcription factors and kinases is a crucial mechanism for limiting atherosclerotic plaque development and promoting stability. In rodent models of vascular injury, exenatide demonstrated its potential to reduce restenosis risk post-angioplasty by minimizing neointimal hyperplasia [38].

#### 2.1.4. Signaling in Monocyte Adhesion and Foam Cell Formation

GLP-1 agonists also target macrophages to prevent the formation of lipid-laden foam cells, a hallmark of atherosclerosis. The reduced expression of vascular cell adhesion molecule-1 (VCAM-1) and intercellular adhesion molecule-1 (ICAM-1) by GLP-1 agonists decreases monocyte attachment to endothelial cells, thus preventing the initial process of atherogenesis in ApoE-knockout mice [9,24]. Within the plaque in that model, the activation of ATP-binding cassette transporter A1 (ABCA1) in macrophages leads to enhanced cholesterol efflux, which prevents foam cell development [24].

### 2.2. Endothelial Function and Vascular Homeostasis

The development of CVD is marked by endothelial dysfunction that leads to impaired vasodilation as well as elevated oxidative stress and increased prothrombotic conditions [39]. The following mechanisms explain how GLP-1 agonists improve endothelial function:

#### 2.2.1. Endothelial Nitric Oxide Synthase (eNOS)/Nitric Oxide (NO) Signalling in Endothelial Cells

A primary effect on endothelial cells is the enhanced production of NO. GLP-1agonists increase the phosphorylation and activation of eNOS, a key enzyme for NO production [26]. This activation promotes vasodilation, reduces vascular stiffness, and has anti-thrombotic properties. Research conducted with T2DM patients demonstrated that exenatide led to a 2–3% improvement in flow-mediated dilation, which served as an indicator of better endothelial function [27]. Liraglutide increased eNOS phosphorylation in human endothelial cells, enhancing vascular relaxation [26].

#### 2.2.2. Blood Pressure Reduction

GLP-1 agonists lower systolic blood pressure (SBP) by 2–5 mmHg through natriuresis, reduced sympathetic activity, and improved vascular compliance [9]. The LEADER trial demonstrated that liraglutide treatment resulted in a 1.2 mmHg reduction in systolic blood pressure, which contributed to the overall decrease in MACE [6]. Dulaglutide produced the same blood pressure reduction as observed in REWIND by lowering SBP by 1.7 mmHg [8].

#### 2.2.3. Anti-Thrombotic Effects

GLP-1 agonists decrease platelet aggregation and tissue factor expression and therefore lower the likelihood of thrombotic events [40]. Preclinical research showed that liraglutide inhibited thrombus formation by decreasing the expression of plasminogen activator inhibitor-1 (PAI-1) and enhancing fibrinolysis [40]. Liraglutide demonstrated reduced platelet activity in T2DM patients, which could minimize their risk of acute coronary syndrome [41].

#### 2.2.4. Blood–Brain Barrier (BBB) Protection

In cerebrovascular contexts, GLP-1 agonists stabilize BBB integrity by upregulating tight junction proteins and reducing MMP-9 activity, potentially mitigating stroke-related damage and vascular dementia pathology [13]. Exenatide demonstrated the ability to reduce BBB permeability in rodent stroke models [13].

### 2.3. Anti-Inflammatory Properties

The progression of CVD includes atherosclerosis as well as heart failure and cerebrovascular disease because of chronic inflammation [42]. The anti-inflammatory properties of GLP-1 agonists are very potent in their therapeutic application.

#### 2.3.1. Cytokine Modulation

The reduction of pro-inflammatory cytokines (e.g., TNF-α, IL-6, IL-1β) and the elevation of anti-inflammatory cytokines (e.g., IL-10, adiponectin) occur through their action [9,10]. Semaglutide led to a 20–30% decrease in high-sensitivity C-reactive protein (hs-CRP) levels during CVOTs because of its systemic anti-inflammatory impact [28]. T2DM patients experienced a 15% reduction in their IL-6 levels after taking liraglutide [28].

#### 2.3.2. Signaling in Macrophages and Other Immune Cells 

A key mechanism within macrophages is the suppression of the NOD-, LRR- and pyrin domain-containing protein 3 (NLRP3) inflammasome [29]. By inhibiting this pathway, GLP-1 agonists curb the release of potent pro-inflammatory cytokines IL-1β and IL-18 [29]. The inflammatory pathways that lead to tissue damage become more severe because NLRP3 activation plays a key role in both atherosclerosis and heart failure [29]. Exenatide decreased NLRP3 expression in human macrophages, which resulted in reduced inflammatory processes [29].

#### 2.3.3. Macrophage Polarization

GLP-1 agonists promote macrophages to change their inflammatory status from pro-inflammatory (M1) to anti-inflammatory (M2), which reduces inflammation in both the blood vessels and heart [43]. Through its action on human monocytes, liraglutide stimulated M2 polarization, which resulted in reduced atherosclerosis progression [43].

#### 2.3.4. Microglial Activation

Research on stroke and vascular dementia in animals has shown that GLP-1 agonists reduce both microglial activation and neuroinflammation, which leads to better protection of the brain from additional injury and cognitive deterioration [13]. The administration of semaglutide in rodent stroke models decreased NLRP3 expression in microglial cells, which resulted in better neurological recovery [13].

### 2.4. Direct Cardioprotective Effects

The activation of GLP-1 receptors in cardiomyocytes results in direct cardioprotective effects, which are particularly evident in conditions of ischemia and heart failure. 

#### 2.4.1. Pro-Survival Signalling in Cardiomyocytes: Myocardial Ischemia Protection

In preclinical models of myocardial ischemia, the activation of GLP-1 receptor agonists reduces infarct size by activating powerful pro-survival signalling cascades within the cardiomyocyte. These include the phosphatidylinositol 3-kinase (PI3K)/Akt and PKA pathway [9,30]. The activation of these well-known pro-survival kinases highlights a direct, insulin-independent cardioprotective mechanism that protects cardiomyocytes from ischemic injury. For instance, the administration of exenatide resulted in a 20–25% decrease in infarct size in porcine MI models by increasing the levels of cAMP [31]. Semaglutide activated Akt signalling in ischemic mouse hearts, reducing apoptosis [30].

#### 2.4.2. Anti-Apoptotic and Anti-Oxidative Effects

The drugs activate anti-apoptotic proteins and block pro-apoptotic pathways to protect cardiomyocytes against ischemia and oxidative stress [30]. Oxidative stress, a key mediator of cardiovascular damage in diabetes and obesity, is significantly attenuated by GLP-1 agonists through multiple mechanisms [32]. GLP-1 agonists reduce mitochondrial reactive oxygen species (ROS) production by preserving mitochondrial membrane potential and enhancing the efficiency of the electron transport chain [33]. They upregulate the expression and activity of key antioxidant enzymes, including manganese superoxide dismutase (MnSOD), catalase, and glutathione peroxidase-1 (GPx-1), through activation of the Nrf2 (nuclear factor erythroid 2-related factor 2) pathway [44]. Liraglutide treatment increased Nrf2 nuclear translocation by 2.5-fold and enhanced antioxidant response element (ARE)-mediated gene transcription in cardiomyocytes exposed to high glucose [34]. Furthermore, GLP-1 agonists reduce Nicotinamide Adenine Dinucleotide Phosphate (NADPH) oxidase activity, a major source of cardiovascular ROS. Semaglutide decreased NADPH oxidase 4 (NOX4) expression by 40% in endothelial cells and reduced superoxide production by 35% in vascular smooth muscle cells [45]. This reduction in oxidative stress translates to decreased lipid peroxidation, protein carbonylation, and DNA damage, ultimately preserving cellular function and viability [46]. Dulaglutide prevented the death of cardiomyocytes in rodent HF models by maintaining cardiac function and reducing oxidative stress markers by 30–40% [30].

#### 2.4.3. Myocardial Metabolism

The GLP-1 agonists increase myocardial glucose uptake and change the metabolic pathways toward glucose oxidation by increasing the energy efficiency of the heart during ischemia or failure [9]. The administration of semaglutide to HFpEF models resulted in an increase in cardiac ATP production that led to an improvement in heart contractility [11]. The administration of liraglutide to diabetic cardiomyopathy models led to increased fatty acid oxidation, which reduced lipotoxicity [9].

#### 2.4.4. Anti-Fibrotic Effects

The drugs inhibit the transforming growth factor-beta (TGF-β) signalling pathway, which reduces myocardial fibrosis and leads to the preservation of ventricular function in animal models [30]. This anti-fibrotic action is crucial for preventing pathological cardiac remodelling. The administration of dulaglutide reduced cardiac remodelling in rodent HF models. Semaglutide treatment in obese mice led to decreased myocardial collagen levels, which resulted in better diastolic function [30].

### 2.5. Weight Loss and Metabolic Benefits

Obesity is a major CVD risk factor, and GLP-1 agonists promote significant weight loss (5–15% of body weight) by reducing appetite, enhancing satiety, and slowing gastric emptying [47]. Weight loss leads to decreased inflammation in the body, increased insulin sensitivity and reduced mechanical stress on the cardiovascular system [47]. The STEP trials showed that semaglutide (2.4 mg weekly) caused a weight loss of 12–15% in obese patients and decreased hs-CRP levels, improved lipid profiles and lowered blood pressure [48]. Weight loss has been proven to decrease the amount of visceral fat that is accumulated which is a key factor in the development of atherosclerosis and HFpEF [49]. Additionally, GLP-1 agonists enhance insulin signaling in the periphery and in the brain which may improve vascular dementia through improved glucose metabolism in the brain and decreased neuroinflammation [13].

### 2.6. Neuroprotective Effects in Cerebrovascular Disease

GLP-1 agonists exhibit neuroprotective effects relevant to stroke and vascular dementia through several mechanisms demonstrated in preclinical studies.

#### 2.6.1. Cerebral Blood Flow (CBF) Improvement

The compounds increase CBF through their ability to cause vasodilation and decrease vascular resistance, which was observed in rodent stroke models [13]. Exenatide decreased the size of the infarct in models of ischemic stroke by improving cerebral blood flow [13].

#### 2.6.2. Anti-Oxidative Stress

GLP-1 agonists reduce reactive oxygen species (ROS) and upregulate antioxidant enzymes, protecting neurons from ischemic damage [13].

#### 2.6.3. Synaptic Protection and Neurogenesis

They enhance synaptic plasticity and hippocampal neurogenesis, potentially mitigating cognitive decline in vascular dementia [13]. Liraglutide was found to increase brain-derived neurotrophic factor (BDNF) in rodent vascular dementia models leading to improved memory [50].

### 2.7. Interaction and Convergence of Signalling Pathways

**Figure 2:**
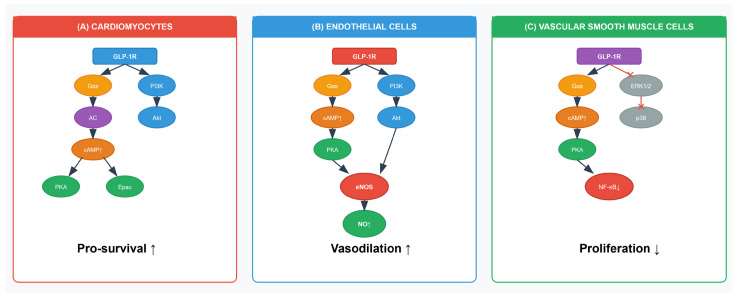
Cell Type-Specific Signaling Pathways of GLP-1 Receptor Agonists in Cardiovascular Protection. Schematic representation of the molecular signaling cascades activated by GLP-1 receptor (GLP-1R) stimulation in three major cardiovascular cell types. (**A**) Cardiomyocytes: GLP-1R activation triggers dual signaling pathways: (i) Gαs-mediated activation of adenylyl cyclase (AC) leading to increased cAMP production and subsequent activation of protein kinase A (PKA) and exchange protein activated by cAMP (Epac), and (ii) PI3K/Akt pathway activation. These converging pathways ultimately promote cardiomyocyte survival (Pro-survival ↑). (**B**) Endothelial cells: GLP-1R stimulation activates both cAMP/PKA and PI3K/Akt signaling cascades. The PI3K/Akt pathway phosphorylates endothelial nitric oxide synthase (eNOS), resulting in increased nitric oxide (NO) production. PKA activation also contributes to eNOS phosphorylation. These pathways collectively enhance vasodilation (Vasodilation ↑). (**C**) Vascular smooth muscle cells (VSMCs)**:** GLP-1R activation in VSMCs primarily leads to Gαs-mediated cAMP/PKA signaling while simultaneously inhibiting pro-proliferative pathways including ERK1/2 and p38 MAPK (indicated by inhibitory symbols). PKA activation results in suppression of NF-κB signaling. These mechanisms collectively reduce VSMC proliferation (Proliferation ↓), contributing to the anti-atherosclerotic effects of GLP-1 agonists. Arrows indicate activation pathways; blunt-ended lines represent inhibitory signals. The distinct signaling profiles across cell types underscore the pleiotropic cardiovascular protective effects of GLP-1 receptor agonists.

It is critical to recognize that the mechanisms described above are not isolated but are highly interactive and convergent. The true therapeutic efficacy of GLP-1 agonists arises from this multi-pronged attack on CVD pathophysiology [9,10]. Firstly, the anti-inflammatory actions are intrinsically linked to the anti-atherogenic effects. The suppression of the NLRP3 inflammasome and the promotion of an M2 phenotype in macrophages (Section 2.3) directly translates into more stable atherosclerotic plaques with a reduced inflammatory burden (Section 2.1) [29,43]. This interaction highlights that reducing inflammation within the vessel wall is a primary mechanism by which GLP-1 agonists halt atherosclerosis [24,25]. Secondly, improved endothelial function is a prerequisite for preventing atherosclerosis. The ability of GLP-1 agonists to reduce the expression of adhesion molecules, such as VCAM-1 on endothelial cells (Section 2.2) directly prevents the initial infiltration of monocytes into the vessel wall, a key initiating event of atherosclerosis (Section 2.1) [9,26,39]. Finally, common upstream signals, such as cAMP have diverse, cell-specific downstream effects. For example, an increase in cAMP in VSMCs leads to an anti-proliferative effect by inhibiting MAPK/NF-κB (Section 2.1), whereas in cardiomyocytes, it contributes to a pro-survival effect via PKA activation (Section 2.4) [9,30]. This illustrates the elegant complexity of GLP-1RA signalling, where a single class of drugs can exert distinct, beneficial effects in different cell types within the cardiovascular system, leading to a comprehensive protective profile (Figure 2) [10,23].

## 3. Clinical Evidence from Cardiovascular Outcome Trials 

**Figure 3:**
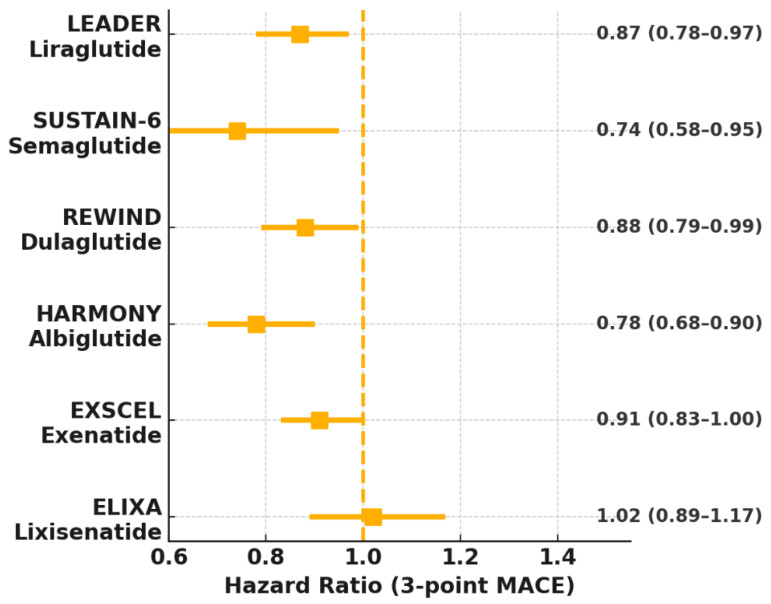
Forest-plot summary of major cardiovascular outcome trials evaluating GLP-1 receptor agonists. Squares denote hazard ratios (HR) for the primary three-point major adverse cardiovascular event (MACE) composite; horizontal bars indicate 95% confidence intervals (CI). Values to the right of each bar provide the exact HR (CI). The dashed vertical line (HR = 1.0) represents no effect; points left of this line favor GLP-1 therapy. Trials are ordered chronologically. ELIXA reports a 4-point MACE.

The cardiovascular benefits of GLP-1 agonists have been extensively studied in CVOTs, mainly in T2DM patients with an elevated risk of cardiovascular events. The trials provide robust evidence of efficacy and safety, with additional insights from meta-analyses and real-world studies (Figure 3 and Table 3 and Table 4) [51].

### 3.1. Liraglutide

The Liraglutide Effect and Action in Diabetes: Evaluation of Cardiovascular Outcome Results (LEADER) trial (2016) enrolled 9340 T2DM patients with high CVD risk [6]. Liraglutide (1.8 mg daily) reduced MACE by 13% (HR 0.87, 95% CI 0.78–0.97) compared to placebo, driven by reductions in cardiovascular death (HR 0.78), non-fatal MI (HR 0.86), and non-fatal stroke (HR 0.89) [6]. It also lowered all-cause mortality (HR 0.85) and achieved modest reductions in SBP (1.2 mmHg), body weight (2–3 kg), and HbA1c (0.4%) [6]. The study demonstrated that patients of all age groups, sexes and levels of CVD risk received equal benefits from the drug, with patients who had experienced cardiovascular events experiencing a 17% decrease in MACE [6].

### 3.2. Semaglutide

The Semaglutide Unabated Sustainability in Treatment of Type 2 Diabetes (SUSTAIN-6) trial (2016) showed a 26% reduction in MACE (HR 0.74, 95% CI 0.58–0.95) in subcutaneous semaglutide (0.5 or 1.0 mg weekly) in 3,297 T2DM patients [7]. The reduction was primarily driven by a 39% decrease in non-fatal stroke (HR 0.61), with trends toward reduced MI (HR 0.74) and cardiovascular death (HR 0.98) [7]. In the STEP-HFpEF trial (2023), semaglutide (2.4 mg weekly) was tested in HFpEF patients with obesity. The results showed that semaglutide improved symptoms (Kansas City Cardiomyopathy Questionnaire [KCCQ] score +16.6 points), physical function (6-minute walk distance +20 meters) and reduced body weight by 13% in obese patients with HFpEF, regardless of T2DM status [11]. The STEP-HFpEF DM trial confirmed similar benefits in T2DM patients [56]. The SELECT trial (2023) in non-diabetic patients with obesity and established CVD reported a 20% MACE reduction (HR 0.80, 95% CI 0.72–0.90), driven by reductions in MI and stroke, marking a paradigm shift for GLP-1 agonists in primary prevention [12]. In the SOUL trial, oral semaglutide showed a 14% MACE reduction (HR 0.86, 95% CI 0.77–0.96) [22].

### 3.3. Dulaglutide

The Researching Cardiovascular Events with a Weekly Incretin in Diabetes (REWIND) trial (2019) evaluated dulaglutide (1.5 mg weekly) in 9901 T2DM patients with a broader risk profile, including 31% with prior CVD [8]. Dulaglutide reduced MACE by 12% (HR 0.88, 95% CI 0.79–0.99), with consistent benefits across MI (HR 0.87), stroke (HR 0.86), and cardiovascular death (HR 0.91) [8]. Its once-weekly dosing and favorable safety profile enhance clinical utility [8]. Subgroup analyses showed greater benefits in patients with prior CVD (HR 0.83) versus primary prevention (HR 0.93) [8]. Dulaglutide also reduced renal events, highlighting its cardio-renal benefits [57].

### 3.4. Other GLP-1 Agonists

- **Exenatide:** The EXSCEL trial (2017) showed a non-significant 9% MACE reduction (HR 0.91, 95% CI 0.83–1.00) in 14,752 T2DM patients, suggesting modest cardiovascular benefits [17]. However, it reduced all-cause mortality in some subgroups [17].
- **Albiglutide:** The HARMONY Outcomes trial (2018) reported a 22% MACE reduction (HR 0.78, 95% CI 0.68–0.90) in 9463 T2DM patients, driven by reduced MI [18].
- **Efpeglenatide:** The AMPLITUDE-O trial (2021) demonstrated a 27% MACE reduction (HR 0.73, 95% CI 0.58–0.92) in 4076 T2DM patients, with significant reductions in stroke and HF events [19].
- **Lixisenatide:** The ELIXA trial (2015) showed no significant MACE reduction (HR 1.02, 95% CI 0.89–1.17), indicating heterogeneity among GLP-1 agonists [20].

### 3.5. Heart Failure Outcomes

GLP-1 agonists show promise in heart failure, particularly HFpEF, as follows:

- **STEP-HFpEF and STEP-HFpEF DM:** Semaglutide improved KCCQ scores (+16.6 points), reduced HF hospitalizations (HR 0.79), and enhanced physical function in HFpEF patients with or without T2DM [11,46]. Benefits were attributed to weight loss, reduced inflammation, and improved myocardial metabolism [11].
- **FLOW Trial:** In T2DM patients with chronic kidney disease (CKD), semaglutide reduced the composite of kidney-disease progression or HF events by 27% (HR 0.73, 95% CI 0.54–0.98) alongside renal benefits [58].
- **FIGHT and LIVE Trials:** Liraglutide showed neutral effects in heart failure with reduced ejection fraction (HFrEF), highlighting the need for further research in this phenotype [59,60].

### 3.6. Meta-Analyses and Real-World Evidence

A 2021 meta-analysis of major CVOTs confirmed a 14% MACE reduction (HR 0.86, 95% CI 0.80-0.93) with GLP-1 agonists, with significant reductions in MI, stroke, cardiovascular death, and all-cause mortality [51]. Subgroup analyses showed greater benefits in patients with established CVD versus primary prevention [51]. Real-world studies using electronic health records corroborated these findings, reporting 10–15% reductions in CVD events and HF hospitalizations in diverse T2DM populations [61]. Observational data also suggest reduced revascularization rates and improved renal outcomes, reinforcing the cardio-renal-metabolic benefits of GLP-1 agonists [61].

### 3.7. Critical Appraisal of CVOTs and Evidence Gaps

While the collective evidence from the CVOTs outlined in Table 1 is compelling, a critical appraisal reveals important heterogeneity and outstanding questions that are crucial for clinical interpretation [62]. The trials differed significantly in their design, including baseline patient characteristics (e.g., the REWIND trial included a majority of patients for primary prevention, whereas others focused on high-risk secondary prevention populations), follow-up duration, and specific drug properties, all of which likely contribute to the variable magnitude of benefit observed [63]. 

Notably, the ELIXA (lixisenatide) and EXSCEL (exenatide once weekly) trials yielded neutral results for their primary MACE endpoints, underscoring that cardiovascular benefit is not a uniform “class effect” [64]. The lack of benefit with lixisenatide is likely attributable to its shorter half-life and more modest effects on glycemia and weight compared to longer-acting agonists. For EXSCEL, while the point estimate for MACE reduction was favourable (HR 0.91), it did not reach statistical significance, a result potentially confounded by high rates of treatment discontinuation and the inclusion of a relatively lower-risk population [17,65]. These neutral trials highlight that a drug’s specific pharmacokinetic and pharmacodynamic properties are critical determinants of its cardiovascular efficacy. 

A central, ongoing debate is the degree to which the observed cardiovascular benefits are driven by profound weight loss versus weight-independent (pleiotropic) effects of GLP-1 agonism [9]. Weight loss is undeniably a major contributor, as it improves blood pressure, lipids, glycemic control, and systemic inflammation. However, statistical mediation analyses from trials such as SELECT suggest that only a portion of the MACE reduction is explained by weight loss alone, pointing to direct, weight-independent vascular and cardiac benefits [12,66]. When benchmarked against other weight loss interventions, the efficacy of newer agents is remarkable; for example, the 24.2% weight loss seen with retatrutide in a Phase 2 trial approaches results from bariatric surgery [67]. A critical area for future research will be head-to-head trials comparing the long-term cardiovascular outcomes of these different powerful weight-loss modalities to fully dissect their mechanisms of benefit.

## 4. Emerging and Combination Therapies

The success of dual agonism has spurred the development of even more advanced combination therapies targeting multiple metabolic pathways.

### 4.1. Dual GLP-1/GIP Agonists

Dual GLP-1/GIP agonists, such as tirzepatide, represent a significant advancement in incretin-based therapies, offering enhanced efficacy over GLP-1 mono-agonists due to synergistic effects on glucose metabolism, weight loss, and cardiovascular risk factors [14,52]. GIP, another incretin hormone, enhances insulin secretion and modulates fat metabolism, complementing GLP-1’s actions [52]. Tirzepatide, approved for T2DM and obesity, achieves data-proven superior glycemic control (HbA1c reductions of 2.0–2.5%) and weight loss (15–20% of body weight) compared to GLP-1 agonists, such as semaglutide [14]. The SURPASS-CVOT trial evaluated tirzepatide in 13,299 T2DM patients with high CVD risk. The trial demonstrated that tirzepatide was non-inferior to dulaglutide for the primary MACE outcome (HR 0.92; 95% CI, 0.83–1.01). While the trial was primarily a non-inferiority study, a secondary analysis for superiority also showed a significant reduction in time to all-cause death [21]. Tirzepatide also improved lipid profiles (LDL-C reduction by 10–12%) and reduced visceral fat, key drivers of atherosclerosis [14]. Preclinical studies suggest that dual GLP-1/GIP agonism enhances endothelial function, reduces vascular inflammation, and promotes plaque stability more effectively than GLP-1 alone, potentially via amplified cAMP signalling and GIP-mediated anti-lipolytic effects [52]. In HFpEF models, tirzepatide reduced myocardial fibrosis and improved diastolic function, suggesting potential benefits in heart failure [52]. Its anti-inflammatory effects, including reduced hs-CRP and IL-6 levels, further contribute to cardiovascular protection [14]. Real-world data indicate that tirzepatide lowers blood pressure and improves renal function, supporting its role in the cardio–renal–metabolic axis [61]. However, challenges include higher costs and limited long-term safety data, necessitating further studies to confirm its cardiovascular benefits in non-diabetic populations and cerebrovascular conditions, such as vascular dementia [15]. Dual GLP-1/GIP agonists represent a significant new strategy for cardiovascular risk reduction, with ongoing research exploring triple GLP-1/GIP/glucagon agonists for even greater efficacy [68].

### 4.2. CagriSema (Cagrilintide/Semaglutide)

This combination contains semaglutide which functions as a GLP-1 agonist and cagrilintide which is a long-acting amylin analogue. The pancreas secretes amylin together with insulin to activate central satiety pathways independently from GLP-1. The Phase 2 clinical trial of CagriSema which combined cagrilintide and semaglutide led to 15.6% weight reduction in T2DM patients while achieving superior glycemic control and better outcomes than each drug alone [53,54]. The combined treatment of incretin and amylin pathways shows potential to deliver better metabolic and weight-loss benefits and cardiovascular risk reduction advantages though cardiovascular outcome data remain limited.

### 4.3. Retatrutide (GLP-1/GIP/Glucagon Triple Agonist)

The development of retatrutide represents a major breakthrough because it now targets the glucagon receptor alongside GLP-1 and GIP receptors. While GLP-1 receptor suppresses glucagon release, the concurrent activation of the glucagon receptor simultaneously increases energy expenditure and enhances weight loss and hepatic fat metabolism. A Phase 2 trial conducted on obese patients showed that retatrutide produced weight loss of 24.2% after 48 weeks, a result that approaches outcomes seen with bariatric surgery [55]. It also led to substantial improvements in both blood pressure measurements and lipid profiles. While these multi-agonist molecules demonstrate significant potential, their long-term cardiovascular outcome trials have not yet been completed, and long-term safety remains to be established [55].

## 5. Applications in Specific Cardiovascular Conditions

### 5.1. Atherosclerotic Cardiovascular Disease (ASCVD)

GLP-1 agonists function as treatment recommendations for T2DM patients who have ASCVD according to CVOT data that demonstrates reduced incidence of MI and stroke [4]. Their anti-atherogenic effects together with plaque stabilization capabilities along with decreased vascular inflammation and enhanced lipid profiles make them an ideal choice for secondary prevention [9,24]. The cerebrovascular protective benefits of semaglutide became evident through the 39% stroke reduction observed in SUSTAIN-6 [7]. Liraglutide’s effectiveness in reducing myocardial infarction rates in LEADER trial demonstrated its benefits for patients with coronary artery disease [6]. Real-world evidence shows GLP-1 agonists lower the need for percutaneous coronary interventions and other revascularization procedures especially in patients who have already had an MI [61]. The use of liraglutide and semaglutide in clinical imaging has shown their ability to slow down atherosclerosis development according to carotid intima-media thickness tests [37].

### 5.2. Heart Failure

HF and its specific subtype HFpEF represent two significant therapeutic areas where GLP-1 agonists are being developed. Patients with obese HFpEF who had T2DM or not received treatment with GLP-1 agonists in STEP-HFpEF trials which showed KCCQ score improvements of +16.6 points and weight loss of 13% and reduced HF hospitalization rates by 79% [11,56]. The therapeutic benefits stem from weight reduction and decreased epicardial fat together with enhanced myocardial energy metabolism and reduced systemic inflammation [11]. Clinical research on HFrEF patients shows that GLP-1 agonists enhance left ventricular ejection fraction (LVEF) while reducing fibrosis according to preclinical investigations although clinical trial data for liraglutide remains neutral in FIGHT and LIVE [59,60]. The FLOW trial demonstrated a 27% decrease in heart failure events among T2DM patients with CKD thus confirming their effectiveness in HF management [58].

### 5.3. Stroke and Cerebrovascular Disease

The two GLP-1 agonists have proven to lower ischemic stroke risk through the SUSTAIN-6 (HR 0.61) and REWIND (HR 0.86) trials [7,8]. The neuroprotective effects of GLP-1 agonists in rodent models include enhanced cerebral blood flow and minimized oxidative stress and BBB stabilization mechanisms [13]. The preclinical research with liraglutide showed better stroke outcomes alongside reduced stroke severity and improved functional results because of its ability to increase vascular endothelial growth factor (VEGF) expression [13]. Research with semaglutide demonstrated its potential to prevent additional strokes because it improved neurological recovery in animal models of ischemic stroke [13].

### 5.4. Vascular Dementia and Cognitive Impairment

Vascular dementia develops from cerebrovascular damage through endothelial dysfunction and persistent inflammation and white matter damage [13,50,69]. Vascular dementia presents promising therapeutic opportunities for GLP-1 agonists because they:

#### 5.4.1. Reducing White Matter Injury

Research shows that GLP-1 agonists safeguard oligodendrocytes as they decrease white matter hyperintensities in preclinical studies thus maintaining neural connections [13].

#### 5.4.2. Enhancing Neurogenesis

Liraglutide stimulated new hippocampal neuron growth in rodent vascular dementia experiments which led to enhanced memory performance and better executive abilities [50].

#### 5.4.3. Anti-Inflammatory Effects 

The reduction of microglial activation together with lowered pro-inflammatory cytokines by GLP-1 agonists helps prevent neuroinflammation which drives vascular dementia in preclinical studies [13].

### 5.5. Non-Diabetic Populations

The SELECT trial established a new standard by showing GLP-1 agonists reduced MACE by 20% among non-diabetic patients who had obesity along with cardiovascular disease [12]. The therapeutic advantages of GLP-1 agonists emerge from weight reduction of 12–15% along with decreased hs-CRP levels and enhanced endothelial function [12]. Clinical data from semaglutide-treated non-diabetic obese patients confirm its effectiveness in lowering cardiovascular events [61,70].

### 5.6. Cardio–Renal–Metabolic Axis

The cardio-renal-metabolic axis represents a vital therapeutic objective since T2DM, CKD and CVD commonly coexist [70]. The FLOW trial demonstrated that semaglutide decreased kidney disease progression along with cardiovascular events in T2DM patients who had CKD while reducing HF events by 27% [58]. The drug achieves these benefits through decreased albuminuria and enhanced glomerular filtration and body-wide anti-inflammatory responses [58]. GLP-1 agonists offer a comprehensive benefit to the axis which makes them an essential treatment for patients with multiple health conditions [70].

### 5.7. Diabetic Cardiomyopathy

Diabetic cardiomyopathy represents a distinct clinical entity characterized by myocardial dysfunction in diabetic patients independent of coronary artery disease, hypertension, or valvular disease [71]. This condition affects approximately 12–20% of diabetic patients and significantly increases the risk of heart failure and cardiovascular mortality [72]. GLP-1 agonists have emerged as promising therapeutic agents for diabetic cardiomyopathy through multiple protective mechanisms. The pathophysiology of diabetic cardiomyopathy involves metabolic disturbances, oxidative stress, inflammation, and myocardial fibrosis [73]. GLP-1 agonists address these pathophysiological mechanisms through several pathways. First, they improve myocardial glucose metabolism by enhancing insulin sensitivity and glucose uptake while reducing lipotoxicity through decreased fatty acid oxidation [74]. Liraglutide treatment in diabetic cardiomyopathy models demonstrated a 30% reduction in myocardial lipid accumulation and improved mitochondrial function [75]. Second, GLP-1 agonists exert potent anti-oxidative effects in the diabetic myocardium. They reduce ROS production by 40–50% through upregulation of antioxidant enzymes, including superoxide dismutase (SOD), catalase, and glutathione peroxidase [76]. Semaglutide treatment in diabetic rats increased myocardial SOD activity by 35% and reduced malondialdehyde levels, a marker of lipid peroxidation, by 45% [77]. Clinical evidence supporting GLP-1 agonists in diabetic cardiomyopathy is growing. A prospective study of 88 T2DM patients with early diabetic cardiomyopathy showed that liraglutide treatment for 6 months improved left ventricular diastolic function (E/e’ ratio decreased from 12.4 to 9.8) and reduced left ventricular mass index by 8% [78]. Echocardiographic studies demonstrate that GLP-1 agonists improve global longitudinal strain, an early marker of subclinical diabetic cardiomyopathy, by 15–20% [79]. The anti-fibrotic effects are particularly relevant in diabetic cardiomyopathy. GLP-1 agonists suppress TGF-β/Smad signalling, reducing collagen deposition and myocardial stiffness [80]. Dulaglutide treatment in diabetic mice reduced myocardial collagen content by 35% and improved diastolic function parameters [81]. Furthermore, GLP-1 agonists preserve mitochondrial integrity, reduce endoplasmic reticulum stress, and inhibit cardiomyocyte apoptosis through activation of the PI3K/Akt and AMPK pathways [82]. Real-world evidence from a registry of 3865 T2DM patients showed that those treated with GLP-1 agonists had a 28% lower incidence of heart failure hospitalization compared to other glucose-lowering therapies, with the benefit being most pronounced in patients with subclinical diabetic cardiomyopathy detected by elevated NT-proBNP levels [83].

## 6. Challenges, Limitations, and Future Directions

While the therapeutic potential of GLP-1 agonists in cardiovascular medicine is significant, several obstacles, evidence gaps, and safety considerations must be addressed to realize their full global impact and ensure their optimal use.

### 6.1. Safety, Tolerability, and Adverse Effects

While GLP-1 agonists have a well-established benefit profile, a thorough understanding of their safety and tolerability is crucial for clinical practice. The most common adverse effects are gastrointestinal in nature, including nausea, vomiting, and diarrhoea. These are typically dose dependent, occur most frequently upon initiation or dose escalation, and often diminish over time [84]. However, they can be severe enough to lead to treatment discontinuation in 5–10% of patients. For subcutaneous formulations, injection-site reactions are also common, though usually mild [85]. 

Concerns about a potential link to pancreatitis were raised in early observations. While pancreatitis remains a warning on product labeling and a theoretical concern due to shared pathways, large-scale CVOTs and subsequent meta-analyses have not shown a statistically significant increase in the risk of acute pancreatitis compared to placebo [86]. Nonetheless, these agents should be used with caution in patients with a history of pancreatitis.

In contrast to the pancreatitis signal, there is a more consistent observation from clinical trials of an increased risk of gallbladder or biliary disease, particularly cholelithiasis (gallstones) [87]. This is not believed to be a direct drug effect but rather a consequence of the rapid and significant weight loss induced by potent GLP-1 agonists, which can alter bile acid composition and gallbladder motility. Clinicians should be aware of this risk, especially when treating patients with a history of gallbladder disease or those experiencing very rapid weight loss. 

Another important safety consideration that emerged from trials such as SUSTAIN-6 is a signal for the potential for an early worsening of pre-existing diabetic retinopathy [88]. This phenomenon is also not thought to be a direct toxic effect of the drug itself, but rather a known risk associated with rapid and substantial improvement in glycemic control, which can also be observed with the initiation of intensive insulin therapy. This finding underscores the critical importance of baseline ophthalmologic assessment and ongoing monitoring for patients with established retinopathy, particularly when initiating potent glucose-lowering therapies that can cause large and swift reductions in HbA1c. 

Other areas of interest, such as potential long-term effects on bone health and fracture risk, are also subjects of ongoing investigation, as significant weight loss can sometimes be associated with changes in bone density. However, a detailed discussion on this topic falls outside the scope of the cardiovascular and metabolic data reviewed in this article, and awaits results from dedicated studies.

### 6.2. Implementation Challenges: Cost, Access, and Adherence

A formidable barrier to the widespread use of GLP-1 agonists is their high cost, which can exceed $1000 per month in many countries [89]. This severely restricts access, particularly in lower-income settings and within health systems operating under strict budget constraints [90]. This economic challenge limits their global health relevance and exacerbates disparities in cardiovascular care. The future development and availability of biosimilars or generic formulations will be crucial for improving affordability and equitable access. Furthermore, as these therapies are primarily injectable (with the exception of oral semaglutide), ensuring long-term patient adherence can be a challenge, requiring robust patient education, support systems, and simpler delivery devices.

### 6.3. Unanswered Mechanistic and Clinical Questions

Despite extensive research, the precise mechanisms driving all the cardiovascular benefits remain partially unresolved. As discussed (Section 3.7), the relative contributions of profound weight loss versus direct, weight-independent effects on the vasculature, heart, and inflammatory pathways are still being actively debated [91]. Further investigation is also needed in specific patient populations. For instance, while their benefit in HFpEF is established, their role in heart failure with reduced ejection fraction (HFrEF) remains uncertain, with trials such as FIGHT and LIVE showing neutral results [59,60]. The applicability of these agents for primary prevention in non-obese individuals without diabetes is also an important evidence gap.

### 6.4. Future Directions and Precision Medicine

Future research should focus on several key areas. Head-to-head trials comparing different GLP-1 agonists and the newer dual/triple agonists are needed to provide clarity for clinical decision making [92]. There is a pressing need to expand clinical trial evidence to solidify their therapeutic role in non-diabetic populations for primary prevention and for specific conditions, such as vascular dementia. Combination therapies, particularly with SGLT2 inhibitors, hold immense promise for synergistically targeting multiple pathways to reduce residual cardiovascular and renal risk, and this is an active area of investigation [93].

Finally, a shift towards precision medicine could optimize the use of GLP-1 agonists and maximize their benefit-to-risk ratio. This involves moving beyond a “one-size-fits-all” approach to identify which patients will derive the most benefit. A key avenue is biomarker-driven patient selection, which involves identifying patient subgroups based on their underlying pathophysiology [94]. For instance, given the potent anti-inflammatory properties of GLP-1 agonists, which can reduce hs-CRP levels by 20–30% [28], future trials could explore whether patients with a high baseline inflammatory burden (elevated hs-CRP) derive a disproportionately larger cardiovascular benefit [95]. Similarly, patients with a pronounced metabolic phenotype, characterized by high baseline triglycerides, elevated apolipoprotein B, and poor glycemic control, might be ideal candidates, as these are parameters directly and favourably modulated by these agents [36]. The success of trials such as FLOW in patients with chronic kidney disease underscores the value of targeting a cardio–renal phenotype, where biomarkers such as high baseline albuminuria could identify individuals poised to receive dual cardiovascular and renal protection [58].

This biomarker-based approach complements stratification by clinical phenotype. Existing CVOT subgroup analyses already provide a basis for this, consistently showing greater benefits in patients with established ASCVD compared to those in a primary prevention setting [51]. This suggests that the ‘secondary prevention’ phenotype is particularly responsive. Similarly, patients with complex cardio–renal–metabolic disease represent another distinct phenotype where the multifaceted benefits of these agents on the heart, kidneys, and metabolism could be maximally leveraged [70]. Future research should aim to integrate these clinical and biomarker profiles with novel genetic, proteomic, or metabolomic signatures to predict both treatment efficacy and the risk of adverse effects, paving the way for truly personalized incretin-based therapy.

## 7. Conclusions

GLP-1 agonists have become a cornerstone of cardiovascular medicine by showing substantial reductions in MACE and heart failure events and stroke, along with potential benefits for vascular dementia. Their wide range of therapeutic actions that include anti-atherogenic and anti-inflammatory effects, along with endothelial protection and cardioprotective benefits, explain their effectiveness across various cardiovascular disease subtypes. Robust evidence from landmark CVOTs, such as LEADER, SUSTAIN-6, and SELECT has proven GLP-1 agonists serve as essential treatment options for T2DM patients at high cardiovascular risk and indicate their growing applications in HFpEF and non-diabetic patients and cerebrovascular disease. The dual GLP-1/GIP agonist tirzepatide demonstrates potent efficacy, which indicates better potential for cardiovascular risk reduction. However, realizing the full worldwide impact of this therapeutic class requires a sober acknowledgement and concerted effort to address significant challenges, including prohibitive costs, long-term safety monitoring, and critical gaps in our understanding of their mechanisms and optimal use. Future research must focus on developing precision medicine strategies, expanding indications through rigorous trials, and creating accessible and affordable solutions to combat the ever-increasing global burden of CVD and related metabolic disorders.

## Figures and Tables

**Table 1:** Completed cardiovascular (or HF-related) outcome trials with incretin-based therapy.

Trial (Year)	Agent/Dose	Study Population (N)	Primary Outcome (3-Point MACE Unless Stated)	Key CV/HF Findings	Key Ref.
LEADER (2016)	Liraglutide 1.8 mg qd	9340 T2DM + high CV risk	↓ MACE 13% (HR 0.87)	↓ CV death 22%, ↓ all-cause mortality 15%	[6]
SUSTAIN-6 (2016)	Semaglutide 0.5/1 mg qw	3297 T2DM	↓ MACE 26% (HR 0.74)	↓ stroke 39%	[7]
EXSCEL (2017)	Exenatide 2 mg qw	14,752 T2DM	NS 9% MACE↓ (HR 0.91)	↓ all-cause mortality in sub-groups	[17]
HARMONY (2018)	Albiglutide 30–50 mg qw	9463 T2DM + ASCVD	↓ MACE 22% (HR 0.78)	Driven by MI ↓	[18]
REWIND (2019)	Dulaglutide 1.5 mg qw	9901 T2DM (31% ASCVD)	↓ MACE 12% (HR 0.88)	Consistent across MI/stroke	[8]
AMPLITUDE-O (2021)	Efpeglenatide 4–6 mg qw	4076 T2DM	↓ MACE 27% (HR 0.73)	↓ stroke & HF events	[19]
ELIXA (2015)	Lixisenatide 20 µg qd	6068 T2DM + recent ACS	Neutral (HR 1.02)	Heterogeneous class effect	[20]
STEP-HFpEF (2023)	Semaglutide 2.4 mg qw	529 obese HFpEF (±T2DM)	KCCQ +16.6 pts†	↓ HF hospitalisation (HR 0.79)	[11]
SELECT (2023)	Semaglutide 2.4 mg qw	17,604 obese, non-DM + ASCVD	↓ MACE 20% (HR 0.80)	Benefit in primary prevention	[12]
SURPASS-CVOT	Tirzepatide 10/15 mg qw	13,299 T2DM + high CV risk	Non-inferior vs dulaglutide; (HR 0.92)	Secondary analysis → significant-cause death ↓	[21]
SOUL (2025)	Oral semaglutide	9650 T2DM + ASCVD/CKD	↓ MACE 14% (HR 0.86)	Secondary analysis →	[22]

MACE, Major Adverse Cardiovascular Event; CV, Cardiovascular; HF, Heart Failure; T2DM, Type 2 Diabetes Mellitus; ASCVD, Atherosclerotic Cardiovascular Disease; MI, Myocardial Infarction; ACS, Acute Coronary Syndrome; KCCQ, Kansas City Cardiomyopathy Questionnaire; CKD, Chronic Kidney Disease.

**Table 2:** Principal mechanisms underpinning cardiovascular protection by GLP-1 agonists.

Mechanistic Domain	Major Actions	Representative Evidence	Key Ref.
Anti-atherogenic	↓ LDL-C & TG, plaque stabilization, ↓ VSMC proliferation	Liraglutide & semaglutide attenuated atherosclerosis in ApoE/LDLR-KO mice	[24,25]
Endothelial/Vascular	↑ eNOS-NO, improved FMD, ↓ SBP 2–5 mmHg, antithrombotic	Exenatide ↑ FMD 2–3% (*human data*); liraglutide eNOS activation (in vitro *data*)	[26,27]
Anti-inflammatory	↓ TNF-α, IL-6; NLRP3 inhibition; macrophage M2 shift	Semaglutide ↓ hs-CRP 20–30% (*human data*); exenatide ↓ NLRP3 (in vitro *data*)	[28,29]
Direct cardioprotection	↓ Ischemia–reperfusion injury, anti-apoptotic, anti-fibrotic	Exenatide ↓ infarct 20–25% *in porcine models*; semaglutide ↑ Akt (in vitro *data*)	[30,31]
Metabolic/Weight	5–15% weight loss; improved insulin sensitivity	STEP trials: semaglutide 12–15% weight loss (*human data*)	[32,33]
Neuro-/Cerebro-vascular	BBB protection, ↑ CBF, ↑ BDNF; ↓ ROS	Exenatide ↓ BBB permeability (*animal data*); liraglutide ↑ neurogenesis (*animal data*)	[13,34]

GLP-1, Glucagon-Like Peptide-1; LDL-C, Low-Density Lipoprotein Cholesterol; TG, Trigrlyceride; VSMC, Vascular Smooth Muscle Cells, LDLR, Low-Density Lipoprotein Receptor; KO, knockout; eNOS, Endothelial Nitric Oxide Synthase; FMD, Flow-Mediated Dilatation; SBP, Systolic Blood Pressure; NLRP3, NOD-, LRR-, and Pyrin Domain-Containing Protein 3; hs-CRP, High-Sensitivity C-Reactive Protein; BBB, Blood-Brain Barrier; CBF, Cerebral Blood Flow; BDNF, Brain-Derived Neurotrophic Factor; ROS, Reactive Oxygen Species.

**Table 3:** Next-generation incretin-based combination therapies.

Agent/Platform	Mechanism	Development Status	Key Efficacy Signals	Key Ref.
Tirzepatide	Dual GLP-1/GIP	Approved; CVOT (SURPASS-CVOT)	↓ MACE (see Table 1); HbA1c −2.0–2.5%, weight −15–20%	[21,52]
CagriSema (cagrilintide + semaglutide)	Amylin analog + GLP-1	Phase 2 obesity & T2DM trials	↓ Weight loss; favourable glycaemia & lipids	[53,54]
Retatrutide (LY 3437943)	Triple GLP-1/GIP/Glucagon	Phase 2 (obesity 2023); CV studies in design	Up to 24% weight loss; ↑ energy expenditure, lipid lowering; pre-clinical CV benefit	[55]

GLP-1, Glucagon-Like Peptide-1; GIP, Glucose-Dependent Insulinotropic Polypeptide; CVOT, Cardiovascular Outcome Trials; MACE, Major Adverse Cardiovascular Event; T2DM, Type 2 Diabetes Mellitus; CV, Cardiovascular.

**Table 4:** Comparative Profile of Key GLP-1 Receptor Agonists in Major CVOTs.

Feature	Liraglutide (LEADER)	Semaglutide (SUSTAIN-6)	Dulaglutide (REWIND)
Dosing Frequency	Once Daily	Once Weekly	Once Weekly
Primary MACE Reduction	13%	26%	12%
Primary Driver of Benefit	CV Death Reduction	Stroke Reduction	Broadly Consistent
All-Cause Mortality	Significant Reduction (15%)	Neutral	Significant Reduction (in some analyses)
Patient Population	High-Risk T2DM	High-Risk T2DM	Broader-Risk T2DM
Typical Weight Loss	Modest (~2–3 kg)	Moderate (~4–6 kg)	Modest (~2–3 kg)

GLP-1, Glucagon-Like Peptide-1; CVOT, Cardiovascular Outcome Trials; MACE, Major Adverse Cardiovascular Event; T2DM, Type 2 Diabetes Mellitus.

## Abbreviations

CVD	Cardiovascular Diseases
T2DM	Type 2 Diabetes Mellitus
GLP-1	Glucagon-Like Peptide-1
GIP	Glucose-Dependent Insulinotropic Polypeptide
CVOTs	Cardiovascular Outcome Trials
MACE	Major Adverse Cardiovascular Events
MI	Myocardial Infarction
HFpEF	Heart Failure with Preserved Ejection Fraction
ASCVD	Atherosclerotic Cardiovascular Disease
VSMCs	Vascular Smooth Muscle Cells
LDL-C	Low-Density Lipoprotein Cholesterol
HDL-C	High-Density Lipoprotein Cholesterol
MMP-9	Matrix Metalloproteinase-9
MCP-1	Monocyte Chemoattractant Protein-1
LDLR	Low-Density Lipoprotein Receptor
cAMP	Cyclic Adenosine Monophosphate
NF-κB	Nuclear Factor Kappa-B
MAPK	Mitogen-Activated Protein Kinase
VCAM-1	Vascular Cell Adhesion Molecule-1
ICAM-1	Intercellular Adhesion Molecule-1
ATP	Adenosine Triphosphate
ABCA1	ATP-Binding Cassette Transporter A1
eNOS	endothelial Nitric Oxidase Synthase
NO	Nitric Oxide
SBP	Systolic Blood Pressure
PAI-1	Plasminogen Activator Inhibitor-1
BBB	Blood-Brain Barrier
TNF-α	Tumor Necrosis Factor Alpha
IL-6	Interleukin 6
IL-1β	Interleukin 1 Beta
hs-CRP	High-Sensitivity C-Reactive Protein
NLRP3	NOD-, LRR-, and Pyrin Domain-Containing Protein 3
PI3K	Phosphatidylinositol 3-kinase (PI3K)
ROS	Reactive Oxygen Species
MnSOD	Manganese Superoxide Dismutase
GPx-1	Glutathione Peroxidase-1
Nrf2	Nuclear Factor Erythroid 2-related Factor 2
ARE	Enhanced Antioxidant Response
NADPH	Nicotinamide Adenine Dinucleotide Phosphate
NOX	NADPH Oxidase
TGF-β	Transforming Growth Factor Beta
CBF	Cerebral Blood Flow
BDNF	Brain-Derived Neurotrophic Factor
KCCQ	Kansas City Cardiomyopathy Questionnaire
CKD	Chronic Kidney Disease
HFrEF	Heart Failure with Reduced Ejection Fraction
LVEF	Left Ventricular Ejection Fraction
VEGF	Vascular Endothelial Growth Factor
SOD	Superoxide Dismutase
